# Irresponsiveness of two retinoblastoma cases to conservative therapy correlates with up- regulation of hERG1 channels and of the VEGF-A pathway

**DOI:** 10.1186/1471-2407-10-504

**Published:** 2010-09-22

**Authors:** Pina Fortunato, Serena Pillozzi, Angela Tamburini, Liliana Pollazzi, Alessandro Franchi, Agostino La Torre, Annarosa Arcangeli

**Affiliations:** 1 Department of Pediatric Ophthalmology Meyer Hospital of Florence - Viale Pieraccini, 24 - 50139 Florence - Italy; 2 Department of Experimental Pathology and Oncology, University of Florence Viale G.B. Morgagni, 50 - 50134 Florence - Italy; 3 Department of Pediatric Oncohaematology and Domiciliary Care Meyer Hospital of Florence - Viale Pieraccini, 24 - 50139 Florence - Italy; 4 Orthoptist Ophthalmology Assistant, Florence. P.O. box 30 -50032 - Borgo San Lorenzo, Florence - Italy; 5 Department of Human Pathology and Oncology, University of Florence, - 50134 Florence - Italy; 6 Department of Specialistics Surgical Sciences, University of Florence - Viale Morgagni, 85 - 50134 Florence - Italy

## Abstract

**Background:**

Treatment strategies for Retinoblastoma (RB), the most common primary intraocular tumor in children, have evolved over the past few decades and chemoreduction is currently the most popular treatment strategy. Despite success, systemic chemotherapeutic treatment has relevant toxicity, especially in the pediatric population. Antiangiogenic therapy has thus been proposed as a valuable alternative for pediatric malignancies, in particolar RB. Indeed, it has been shown that vessel density correlates with both local invasive growth and presence of metastases in RB, suggesting that angiogenesis could play a pivotal role for both local and systemic invasive growth in RB. We present here two cases of sporadic, bilateral RB that did not benefit from the conservative treatment and we provide evidence that the VEGF-A pathway is significantly up-regulated in both RB cases along with an over expression of hERG1 K^+ ^channels.

**Case presentation:**

Two patients showed a sporadic, bilateral RB, classified at Stage II of the Reese-Elsworth Classification. Neither of them got benefits from conservative treatment, and the two eyes were enucleated. In samples from both RB cases we studied the VEGF-A pathway: VEGF-A showed high levels in the vitreous, the *vegf-a, flt-1, kdr*, and *hif1-α *transcripts were over-expressed. Moreover, both the transcripts and proteins of the hERG1 K^+ ^channels turned out to be up-regulated in the two RB cases compared to the non cancerous retinal tissue.

**Conclusions:**

We provide evidence that the VEGF-A pathway is up-regulated in two particular aggressive cases of bilateral RB, which did not experience any benefit from conservative treatment, showing the overexpression of the *vegf-a*, *flt-1*, *kdr *and *hif1-α *transcripts and the high secretion of VEGF-A. Moreover we also show for the first time that the *herg1 *gene transcripts and protein are over expressed in RB, as occurs in several aggressive tumors. These results further stress the relevance of the VEGF-A pathway in RB and the correlation with hERG1, making aggressive and recurrent RB cases good candidates for antiangiogenesis therapies based on the targeting of VEGF-A.

## Background

Retinoblastoma (RB) is the most common primary intraocular tumor in children, with an incidence of 1 in 15,000 live births. The retinoblastoma protein (Rb) regulates cell cycle progression and suppresses tumorigenesis through the control of E2F transcription factor function, which in turn represents the link between the Rb pathway and the induction of p53-dependent apoptosis [1].

RB can affect one or both eyes, and may show either endophytic or exophytic growth, sometimes with signs of invasiveness. When tumor cells invade the choroid or the optic nerve thus reaching the sclera or the lamina cribrosa, extraocular growth can occur. Through this mechanism, tumor cells can spread to the brain, the surrounding head bones, or soft tissues. In rare cases, RB disseminates in the whole body giving rise to distant metastases in the bone marrow, liver or skeletal system. In any case, invasion of either the choroid or the optic nerve are risk factors for developing metastases [2-6].

Treatment strategies for RB have gradually evolved over the past few decades. There has been a trend away from enucleation and external beam radiation therapy toward focal 'conservative' treatments, involving primary chemoreduction in conjunction with thermotherapy and cryotherapy [7-11]. This is related to earlier detection of the disease, recognition of more effective chemotherapeutic agents, more focused local treatment modalities, and, most importantly, knowledge of the long-term risks of external beam radiotherapy. Furthermore, recent research in the treatment of RB has concentrated on methods of combining chemotherapy with other local treatment modalities. This approach combines the principle of chemotherapeutic debulking in paediatric oncology with conservative focal therapies in ophthalmology. Chemoreduction is currently the most popular treatment strategy for intraocular RB worldwide. Despite the dramatic clinical responses obtained with multiagent systemic chemotherapy regimens, enthusiasm for this treatment approach has been tempered by the potential toxicities of these drugs in the pediatric population [12].

Antiangiogenic therapy has been proposed as a valuable tool for solid tumors, including pediatric malignancies. In particular, RB tumors are excellent targets for antiangiogenic therapy. Numerous studies have demonstrated that the vascular density of a tumor correlates with the onset of metastases and hence to poor outcome. It has been shown that vessel density correlates with both local invasive growth and presence of metastases in RB [13]. These data suggest that angiogenesis plays a pivotal role for both local and systemic invasive growth in RB [12-17]. One of the most potent angiogenic factor is the vascular endothelial growth factor-A (VEGF-A). Hence VEGF-A represents one of the most powerful targets in antiangiogenesis therapy. Indeed in a retrospective study on several RB cases [14] was reported a positive correlation between VEGF-A staining intensity and different clinico-pathological markers of malignancy (mitotic and apoptotic indexes).

Herein we present two cases of sporadic, bilateral RB: both of them were endophitic and characterized by an elevated grade of differentiation. Nevertheless both cases did not benefit from the conservative treatment. We provide evidence that the VEGF-A pathway is significantly up-regulated in both RB cases along with an over expression of hERG1 K^+ ^channels.

## Methods

### VEGF-A secretion

The level of VEGF-A was determined in the vitreous fluid collected during the enucleation, using the DuoSet ELISA Development System (R&D Systems, Wiesbaden, Germany).

### RNA extraction and RT-PCR

Normal and neoplastic tissue from the same patient were homogenized in a guanidinium thiocyanate solution, and total RNA was extracted according to [18]. cDNA was then synthesized from 2 μg of RNA using 200 U reverse transcriptase SuperScript II (Invitrogen, Groningen, The Netherlands), plus 200 μM each of dNTP and 2.5 μM random hexamers, in a 20 μl final reaction volume, for 50 min at 42°C and 15 min at 70°C.

### Real-Time Quantitative PCR (RQ-PCR)

*Vegf-a*, *flt-1*, *kdr*, *gapdh*, *hif-α herg1a *and *herg1b *mRNA were quantified in both normal and neoplastic tissue by RQ-PCR, with the ABI PRISM 7700 Sequence Detection System and the SYBR Green Master Mix Kit (Applied Biosystems, Foster City, CA) as reported in [19]. The primer sequences were as follows:

*Vegf-a *sense CGAAACCATGAACTTTCTGC, antisenseCCTCAGTGGGCACACACTCC;

*hif-α *sense GTCGCTTCGGCCAGTGTG antisense GGAAAGGCAAGTCCAGAGGTG; *flt1, kdr, gapdh, herg1a*, and *herg1b *primer sequences were as reported in [19]. The level of transcripts in the normal retina of each case was taken as 1.

### Immunohistochemistry (IHC)

Tumor specimens were processed for histological analysis and stained with Hematoxylin and Eosin. IHC was performed as previously reported in [20] on 5-μm sections attached to positive-charged microscope slides. After dewaxing and re-hydrating, the specimens were treated with a 1% H_2_O_2 _solution and antigen retrieval was carried out using: a) Proteinase K solution (5 μg/ml in PBS)(Roche, Milan, Italy) for VEGF-A and hERG1 staining; b) microwave treatment in 10 mM Na citrate (pH 6.0) at 700 W for 10 minutes, twice for CD34 and Ki-67 staining. Permeabilization was performed with 0.1% Triton X100 in UltraVBlock (LabVision, Fremont, CA). The antibodies were: anti VEGF polyclonal antibody (Santa Cruz Biotechnology, Santa Cruz, CA; 1:200), anti CD34 monoclonal antibody (Cederlane, Ontario, Canada; 1:100), anti Ki-67 monoclonal antibody (DCS Innovative Diagnostik-Systeme, Hamburg, Germany; 1:100), anti hERG1 monoclonal antibody, clone A12 (Enzo Life Sciences, Lausen, Switzerland; 1:100). Incubation was carried out at 4°C O/N. Detection was carried out with PicTure Plus Kit (Zymed, Milan, Italy) and DAB chromogen solution (Zymed, Milan, Italy), according to the manufacturer's instructions.

For immunostaining analysis, tumors were scored by assessment of the proportion and intensity of stained tumor cells and then scored by a semiquantitative method as reported in [14]. The positive staining is shown as a brown pigment, localised in the cytoplasm of cells. Positively stained cells were counted in 5 randomly selected fields under a magnification of 400×.

To assess cell apoptosis we analyzed DNA breaks in apoptotic nuclei using the FragEL DNA fragmentation Detection Kit (Calbiochem, San Diego, CA, USA).

The apoptotic index (AI,%) and the proliferation index (PI, %) were calculated by counting 100 cells (× 400) using FrageEL method and Ki-67 staining, respectively. Microvessel density was calculated by counting CD34 positive vessels on microscopic field (200× magnification).

## Cases presentation

In this study we describe two cases of RB that came to our observation in the period 2000-2010. At difference from the 15 cases which were examined and cured in the Department of Pediatric Ophthalmology Meyer Hospital of Florence in the same period, the two cases here presented did not benefit from the conservative treatment. Both cases were sporadic, bilateral, endophitic RB, whose histological evaluation revealed an elevated grade of differentiation so that they were classified as Stage II of the Reese-Elsworth Classification.

### Case 1

An 1 year old male with no family history of RB had right leukocoria at presentation. The first examination revealed a solid mass which occupied the vitreous cavity, and caused a great inferior retinal detachment. There was no evidence of vitreous seeding. Intraocular pressure (IOP) elevation was detected in the right eye. The right eye was enucleated and substituted by an hidrossiapatite implant. Histopathologic examination revealed an elevated grade of differentiation of the tumor without infiltration of the optic disc. In the left eye, we observed a little tumor near the inferior vascular arch, at 1 optic disc distance from the fovea, and another little tumor near the superior vascular arch.

Genetic analysis (FISH fluorescence In Situ Hybridization Retinoblastoma DNA Probe 13q14) demonstrated the absence of RB1 gene delection.

**Figure 1:**
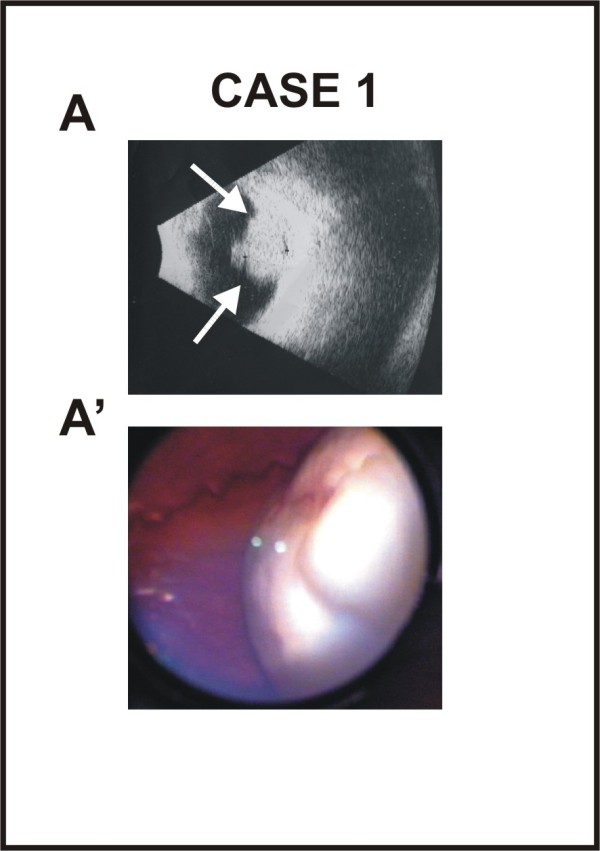
**B scan echography and fundus photograph of the recurrence of case 1 at 5 years follow-up**. (A) B scan echography showing a great tumoral mass with calcification in the superior sector at 5 years follow-up. (A') Fundus photograph showing large tumor mass at 5 years follow-up.

The left eye was treated with chemotherapy associated with focal laser treatment. The AIEOP/RTB-92 Treatment Protocol (4 cycles regimen consisting of Carboplatin (165 mg/kg/die) and VP16 (50 mg/kg/die for 2 days)) was applied. An examination at the end of chemotherapy with B-scan ecography confirmed a partial reduction of both tumor size (from 5,5 mm to less then 4 mm) and thickness (from 2 mm to 1 mm). At the end of chemotherapy, a laser treatment around the tumor mass in the left eye was applied using 450-600 mW, with a spot of 600μ. Subsequently, fundus examination revealed the presence of a tumor in a phase of "cottage cheese" and a ipopigmented atrophic area near the treated areas. Patient was then evaluated every 3 months for the the first year, and every six months starting from the second year. No signs of disease were detected. At the 5 years follow-up, we observed a very aggressive recurrence in a new site at the B-scan ecography (Figure 1A). Fundus examination of the left eye revealed the presence of a new great tumor mass (9,77 mm height and 12 mm at the base) in the superior quadrant, with partial detachment of the surrounding retina (Figure 1A'). This recurrence was treated with chemotherapy (6 cycles of IVad: VCR 1,50 mg/push in 15 ml Sol. Gluc. 5% in 10' Adriblastin 15 mg in 100 ml SF; MESNA 800 mg in 20 ml SF/push; Ifosfamide 2000 mg in 1 vial/20 ml; with preidratation and idratation after the infusion of the chemotherapy) and, subsequently, with cryotherapy. After cryotherapy, we observed a reduction in the tumor size, paralleled by an increased retinal detachment and presence of fibrin in the anterior chamber, that impaired any further examination of the fundus. 30 days after, when the fibrin clot in the anterior chamber was reduced, we observed numerous little new tumors, widespread in the retinal tissue. Given the intraocular dissemination of the tumor, the left eye was enucleated.

### Case 2

**Figure 2:**
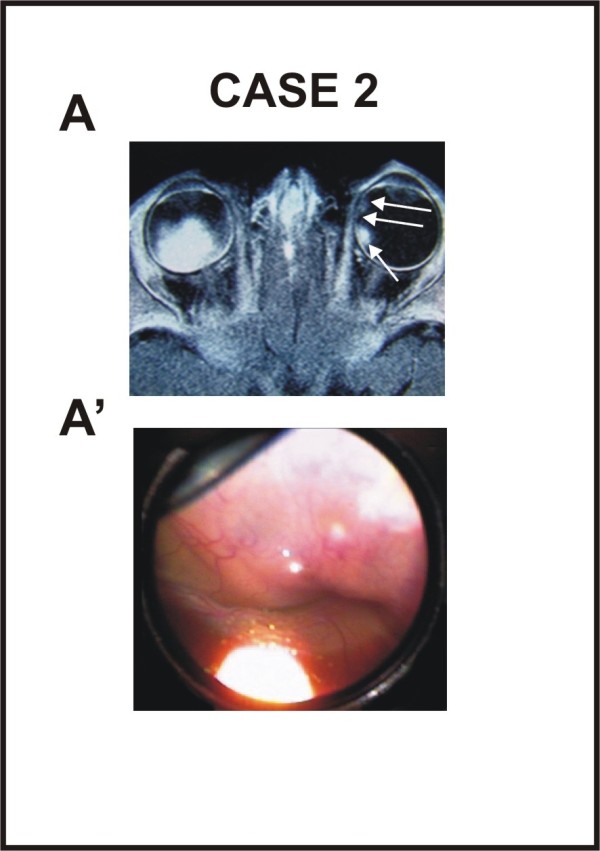
**Nuclear Magnetic Resonance (NMR) and fundus photograph of case 2 at presentation**. (A) NMR evidenced a large calcified mass with no invasion of the optic nerve in the left eye and three tumors of medium and little dimension in the right eye (two of them are very close to each other, so that they seem as a unique mass). (A') Fundus photograph obtained before chemotherapy showing large tumor mass that occupied more than 50% of the vitreous cavity in the left eye.

An 8 months old female with no family history of RB had bilateral leukocoria at presentation. The first examination revealed three masses of medium dimension at 700 μ distance from the fovea, with retinal detachment in the right eye and a mass (whose dimensions were 1.01 × 1.03 × 1.22 cm) in the left eye. The mass occupied 50% of the vitreous cavity and was associated with retinal detachment and vitreal seeding (Figure 2). Nuclear Magnetic Resonance (NMR) showed a large calcified mass with no evidence of invasion of the optic nerve. No metastases were detectable.

The left eye was enucleated according to the AIEOP/RB 05 Treatment Protocol. The histological examination revealed an elevated grade of differentiation of the tumor without infiltration of the optic disc. RB1 gene analysis on peripheral blood lymphocytes demonstrated deletion of exon 24. At the end of the third cycle of chemotherapy the tumors in the right eye were partially reduced (50% of tumor size) and cryotherapy and laser therapy were utilized. Patient was evaluated every 3 months for the the first year, and every six months starting from the second year. Two years later we observed a new aggressive recurrence in the right eye but, unlike Case 1, we decided to treat this patient according to the more recent aquisitions in literature [21]. Therefore, the patient was assigned to receive tandem HDCT/ASCR (high-dose chemotherapy/autologous stem cell rescue). The right eye at the moment (4 months from the end of therapy) shows a modest reduction of dimension of recurrence, between still accompained by a subtotal retinal detachment.

**Figure 3:**
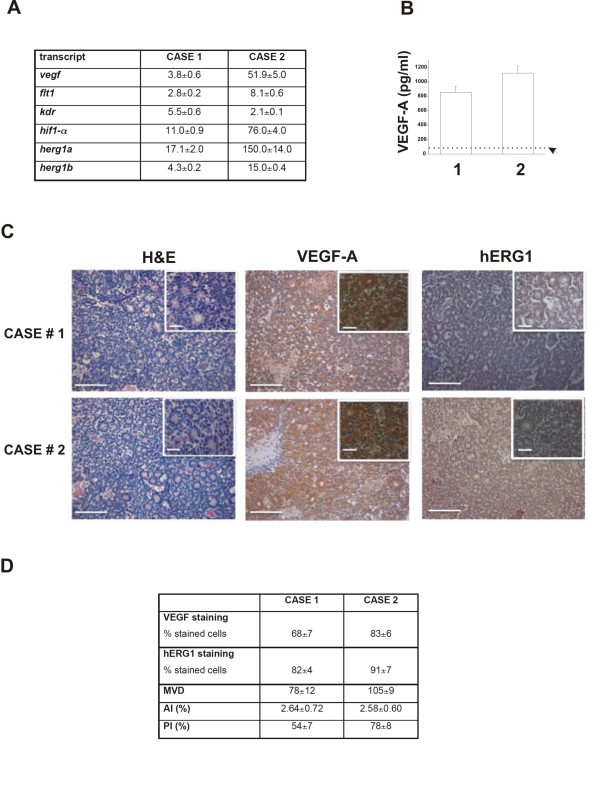
**Analysis of the VEGF-A pathway in the two cases**. (A) mRNA expression levels of *vegf-a*, *flt-1*, *kdr*, *hif-α, herg1a *and *herg1b *in the RB tumor mass. (B) Amount of VEGF-A released in the vitreous fluid by case 1 and case 2. The medium was collected and used for VEGF-A measurement as reported in Methods section. Data are reported as mean ± ESM. Median value relative to control group (as described in 22) is reported (see dotted line). (C) H&E staining (left panels) and immunohistochemistry for VEGF-A (middle panels) and hERG1 proteins (right panels) on case 1 and 2. Inset: magnification of the picture reported in the corresponding panel. Scale bar = 100 μm; Inset scale bar = 50 μm (D) Immunostaining for VEGF-A and hERG1, analysis of microvessel density (MVD) and evaluation of apoptotic and proliferation index (AI and PI respectively). The intensity of VEGF-A and hERG1 staining was also evaluated in normal retinal tissue and the percentage of positive cells was 12,2 ± 2% and 23,08 ± 8% respectively.

In both RB samples we studied the VEGF-A pathway, determining the expression level of the *vegf-a*, VEGF-A receptors (*flt-1 *and *kdr*) and *hif1-α *transcripts. The RQ-PCR technique was applied and results were compared with the expression level of the corresponding normal retina. The *vegf-a *transcript was over-expressed in both cases (3.8 fold increase in case 1; 51.9 fold in case 2). Both *flt-1 *and *kdr *were expressed in both RB samples at levels higher than normal retina (flt1: 2.8 folds in case 1, 8.1 folds in case 2, median value 5.5; kdr: 5.5 folds in case 1, 2.1 folds in case 2, median value 3.8). Finally, the *hif1-a *transcript was over-expressed in both samples (11 fold in case 1), with higher levels in case 2 (76 fold). Both isoforms of the *herg1 *gene (*herg1a *and *herg1b*) were over-expressed compared to the corresponding normal retina. The full length isoform *herg1a *was expressed at higher levels compared to the *herg1b *transcript (*herg1a*: 17.1 folds in case 1, 150 folds in case 2, median value 83.6; *herg1b*: 4.3 folds in case 1, 15 folds in case 2, median value 9.7). Finally, both isoforms were expressed at higher levels in case 2 (Figure 3A).

We then evaluated the levels of VEGF-A in the vitreous fluid obtained during the enucleation of both cases. The levels of VEGF-A were 854 ± 83 pg/ml in case 1 and 1120 ± 98 pg/ml in case 2, respectively (Figure 3B). The median value of VEGF-A was markedly higher in the RB samples (987 pg/ml) when compared to the values of the control group, reported in [22], which refers to aged-paired patients (see Table Two reported in [22]: 59 pg/ml; range 38-135).

Finally, we studied the distribution of the VEGF-A and hERG1 proteins in the two tumor samples by immunohistochemistry. A strong immunopositivity for VEGF-A was detected in both the RB samples analyzed (see Figure 3C): 68 ± 7% and 83 ± 6% of cells were positive for VEGF-A staining, in case 1 and 2, respectively. According to the method reported in [14] most of the cells showed a strong intensity score for both samples (see insets of the VEGF-A panels in Figure 3C). Consistently the microvessel density (MVD), evaluated by CD34 staining, was high, with a significant higher MVD value in case 2 compared to case 1 (p = 0.037, *t *Student test)(Figure 3D). hERG1 expression was evaluated by staining with a monoclonal anti hERG1 antibody (clone A12) which exhibits an high specificity although with a less intense staining (Lastraioli E, manuscript in preparation). 82 ± 4% (in case 1) and 91 ± 7% (in case 2) tumor cells were positive for hERG1, most of them with a moderate intensity score.

Finally, a quantitative analysis of proliferation and apoptosis was performed. The proliferative index (PI) was 54 ± 7% in case 1, and 78 ± 8 in case 2. Similar levels of the apoptic index (AI) were found in both samples, with values comparable to those described in the literature (less than 3%) [23](Figure 3D).

## Conclusion

Due to early diagnosis and improved treatment methods, the survival rate of RB patients has dramatically improved over the last 20 years [24]. In our experience, most RB patients benefit from treatment with conservative therapy. The survival rate dramatically drops to 30% in the cases with presence of metastasis. The presence of metastasis is, therefore, an important feature in the outcome of RB. Pathohistological signs of invasion of eye structures such as the choroid, sclera, and optic nerve anterior or posterior to the lamina cribrosa are highly predictive for the presence of metastasis and correlate with the risk of death [5,6,25]. Data on biological factors influencing the difference in aggressiveness and invasive potential of RB are scarce. The phenomenon of tumor angiogenesis, as an indicator of higher malignant potential, is repeteadly associated with tumor progression and acquirement of malignancy in solid neoplasms. In this scenario, anti-angiogenesis therapy has been tested as a new treatment option has been tested for several tumors, including pediatric malignancies [12,26]. Based on current knowledge, it is believed that patients would profit most from a combination therapy consisting of anti-angiogenic and chemotherapeutic drugs. Such combination therapy targets both the endothelial compartment and the tumor cell compartment, and seems to be more effective in improving the outcome than either therapies alone. Because of the morbidity and potential mortality (from second malignancies), pediatric and ocular oncologists have sought effective alternative methods for treating more aggressive and metastatic RB. Such methods also include anti-angiogenesis therapies targeting the angiogenic growth factor VEGF-A, as a new option. However, a detailed quantitative evaluation of VEGF-A and of the VEGF-A pathway, in aggressive and metastatic RB, is still lacking.

In this manuscript we describe two cases of bilateral RB which did not experience any benefit from conservative treatment options. We provide evidence that the VEGF-A pathway is active in both cases, showing the overexpression of the *vegf-a*, VEGF-A receptors (*flt-1 *and *kdr*) and *hif1-α *transcripts. Moreover, VEGF-A is secreted at high levels in the vitreous of both RB cases, and a strong immunoreaction for VEGF-A can be detected in both RB specimens. Since we previously showed that hERG1 channels regulate *vegf-a *expression and VEGF-A secretion in cancer cells [19,27,28], we also evaluated the expression level of the genes encoding hERG1 channels as well as of the corresponding hERG1 protein in the two RB cases. We here provide evidence, for the first time, that *herg1 *gene isoforms (*herg1a *and *herg1b*) and the hERG1 protein are over expressed in RB as occurred in many different tumors [28]. Finally, both the microvessel density (MVD) and proliferation-related parameters (proliferation index, (PI) and apoptotic index (AI)) were evaluated in both RB cases. It emerged an interesting correlation between the levels of VEGF-A and hERG1 and those of MVD and PI.

These results further stress the relevance of the VEGF-A pathway in RB, making aggressive and recurrent RB cases good candidates for antiangiogenesis therapies based on the targeting of VEGF- A.

## Competing interests

The authors declare that they have no competing interests.

## Authors' contributions

PF carried out the experiments SP carried out the experiments AT designed the study LP carried out the experiments ALT designed the study. AA designed the study and was involved in drafting the manuscript.

All authors read and approved the final manuscript.

## Pre-publication history

The pre-publication history for this paper can be accessed here:

http://www.biomedcentral.com/1471-2407/10/504/prepub
